# Strain-Dependent Differences in Bone Development, Myeloid Hyperplasia, Morbidity and Mortality in Ptpn2-Deficient Mice

**DOI:** 10.1371/journal.pone.0036703

**Published:** 2012-05-08

**Authors:** Florian Wiede, Sock Hui Chew, Catherine van Vliet, Ingrid J. Poulton, Konstantinos Kyparissoudis, Tedjo Sasmono, Kim Loh, Michel L. Tremblay, Dale I. Godfrey, Natalie A. Sims, Tony Tiganis

**Affiliations:** 1 Department of Biochemistry and Molecular Biology, Monash University, Clayton, Victoria, Australia; 2 St. Vincent's Institute of Medical Research, Victoria, Australia; 3 Department of Microbiology and Immunology, University of Melbourne, Parkville, Victoria, Australia; 4 McGill Cancer Centre and Department of Biochemistry, McGill University, Montreal, Quebec, Canada; University Paris Sud, France

## Abstract

Single nucleotide polymorphisms in the gene encoding the protein tyrosine phosphatase TCPTP (encoded by *PTPN2*) have been linked with the development of autoimmunity. Here we have used Cre/LoxP recombination to generate *Ptpn2^ex2−/ex2−^* mice with a global deficiency in TCPTP on a C57BL/6 background and compared the phenotype of these mice to *Ptpn2^−/−^* mice (BALB/c-129SJ) generated previously by homologous recombination and backcrossed onto the BALB/c background. *Ptpn2^ex2−/ex2−^* mice exhibited growth retardation and a median survival of 32 days, as compared to 21 days for *Ptpn2^−/−^* (BALB/c) mice, but the overt signs of morbidity (hunched posture, piloerection, decreased mobility and diarrhoea) evident in *Ptpn2^−/−^* (BALB/c) mice were not detected in *Ptpn2^ex2−/ex2−^* mice. At 14 days of age, bone development was delayed in *Ptpn2^−/−^* (BALB/c) mice. This was associated with increased trabecular bone mass and decreased bone remodeling, a phenotype that was not evident in *Ptpn2^ex2−/ex2−^* mice. *Ptpn2^ex2−/ex2−^* mice had defects in erythropoiesis and B cell development as evident in *Ptpn2^−/−^* (BALB/c) mice, but not splenomegaly and did not exhibit an accumulation of myeloid cells in the spleen as seen in *Ptpn2^−/−^* (BALB/c) mice. Moreover, thymic atrophy, another feature of *Ptpn2^−/−^* (BALB/c) mice, was delayed in *Ptpn2^ex2−/ex2−^* mice and preceded by an increase in thymocyte positive selection and a concomitant increase in lymph node T cells. Backcrossing *Ptpn2^−/−^* (BALB/c) mice onto the C57BL/6 background largely recapitulated the phenotype of *Ptpn2^ex2−/ex2−^* mice. Taken together these results reaffirm TCPTP's important role in lymphocyte development and indicate that the effects on morbidity, mortality, bone development and the myeloid compartment are strain-dependent.

## Introduction

T cell protein tyrosine phosphatase (TCPTP) encoded by *PTPN2* is an intracellular tyrosine-specific protein tyrosine phosphatase (PTP) [1]. TCPTP is expressed ubiquitously, but it is most abundant in cells of hematopoietic origin where it serves as a key regulator of hematopoiesis and immune and inflammatory responses [1]. Genome-wide association studies have linked *PTPN2* single nucleotide polymorphisms (SNPs) with the development of autoimmune diseases including inflammatory bowel disease, type 1 diabetes and rheumatoid arthritis [2], [3], [4]. Recent studies have reported that type 1 diabetes-associated *PTPN2* intronic SNPs may result in decreased *PTPN2* messenger RNA [5]. Moreover, *PTPN2* is deleted in 6% [6] of all T cell acute lymphoblastic leukemias, in particular in those overexpressing the TLX1 transcription factor oncogene, promoting proliferation and enhancing cytokine sensitivity [7]. In addition, alterations in TCPTP expression may be associated with several other malignancies and inflammatory diseases [8], [9], [10], [11].

Several TCPTP substrates have been identified including receptor protein tyrosine kinases (PTKs), such as those for epidermal growth factor [12], [13], [14], insulin [15], [16], [17] and colony stimulating factor-1 [18], cytosolic PTKs, including Janus-activated kinases (JAK)-1 and -3 [19] and c-Src [6], [20], and PTK substrates such the adaptor protein p52^Shc^ [14] and signal transducers and activators of transcription (STAT)-1, -3, 5, and -6 [9], [19], [20], [21], [22], [23]. In keeping with TCPTP being a key regulator of immune and inflammatory responses TCPTP has been shown to attenuate cytokine-induced JAK/STAT signaling in different cell types, including lymphocytes and myeloid cells [18] and to regulate TNF-induced inflammatory responses by attenuating c-Src-induced mitogen-activated protein kinase signaling [6]. More recently, TCPTP has been shown to be important in T cell development and function [24]. Mice that lack TCPTP specifically in T cells exhibit enhanced thymocyte positive selection and enhanced T cell receptor-mediated T cell responses *in vivo* [24].

**Figure 1:**
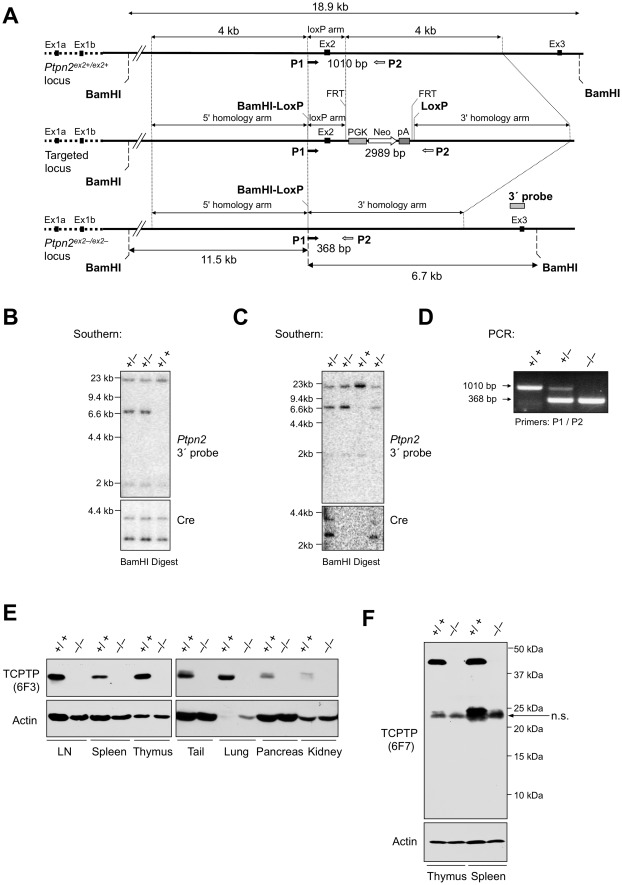
Generation of Ptpn2^ex2+/ex2−^ mice. (**a**) *Ptpn2* genomic locus and targeting design. (**b–c**) Southern blot analysis of *Ptpn2^ex2+/ex2+^* (+/+) and *Ptpn2^ex2+/ex2^* ^−^ (+/−) mice. (**b**) Exon (Ex) 2 floxed mice mated with Oz-Cre mice for the germline deletion of exon 2 and removal of the Neomycin (Neo) resistance cassette. (**c**) Ex2-deleted mice were subsequently mated with C57BL/6 mice for the elimination of the Cre transgene. (**d**) PCR analysis of *Ptpn2^ex2+/ex2+^* (+/+), *Ptpn2^ex2+/ex2^* ^−^ (+/−) and *Ptpn2^ex2^* ^−*/ex2*−^ (−/−) mice. (**e–f**) Immunoblot analysis of TCPTP expression in lymphoid and non-lymphoid tissues detected with antibodies to the TCPTP non-catalytic C-terminus (6F3) and TCPTP N-terminus (6F7; raised against the first 38 residues of TCPTP); a non-specific (n.s.) protein detected by 6F7 is indicated. Results are representative of three independent experiments.

TCPTP's role in hematopoiesis, immunity and inflammation is highlighted by the phenotype of *Ptpn2^−/−^* mice on a mixed BALB/c-129SJ background [25]; mice appear physically normal albeit slightly smaller at 10–14 days of age, but thereon exhibit growth retardation, a hunched posture, piloerection, decreased mobility and diarrhoea and overt signs of wasting disease, succumbing by 3–5 weeks of age [25]. *Ptpn2^−/−^* (BALB/c-129SJ) mice are characterized by widespread mononuclear infiltrates in non-lymphoid tissues and elevated serum interferon (IFN) γ levels by 19 days of age [26]. Consistent with the inflammatory phenotype, *Ptpn2^−/−^* (BALB/c-129SJ) mice are more susceptible to endotoxic shock and macrophages are hypersensitive to lipopolysaccharide [26] and IFNγ [19], whereas *Ptpn2^+/−^* (BALB/c-129SJ) mice are hypersensitive to dextran sulphate sodium (DSS) and the development of colitis [27]. *Ptpn2^−/−^* (BALB/c-129SJ) mice exhibit defects in hematopoiesis, in particular in B cell development and erythropoiesis and develop severe anemia [25]. The defect in B cell development is thought to be attributable, at least in part, to a bone marrow stromal defect and the abnormal secretion of IFNγ by bone marrow stromal cells [28]. *Ptpn2^−/−^* (BALB/c-129SJ) mice develop thymic atrophy associated with a drop in double positive (CD4+CD8+) thymocytes, lymphadenopathy and splenomegaly, the latter being associated with an accumulation of myeloid cells and the sequestration of red blood cells [25].

The *Ptpn2^−/−^* (BALB/c-129SJ) mice were generated more than ten years ago using a homologous recombination approach that resulted in the deletion of approximately 9 kb of genomic sequence, including 1.5 exons (encoding residues 64–121) from the TCPTP catalytic domain, and inserted a Neomycin (Neo) resistance cassette under the control of a Herpes simplex thymidine kinase promoter for the purposes of ES cell selection [25]. It is now widely appreciated that the insertion of Neo genes can have unintended consequences and reduce the expression of nearby flanking genes and complicate or even confound our understanding of gene function [29], [30]. With the advent of Cre/LoxP recombination, constructs can now be designed for the elimination of Neo resistance genes. Given the importance of *PTPN2* in human disease and the complex phenotype in *Ptpn2^−/−^* (BALB/c-129SJ) mice, we used Cre/LoxP gene targeting to generate *Ptpn2* (C57BL/6) null mice without the Neo cassette and compared the phenotype of these mice to that of *Ptpn2^−/−^* (BALB/c-129SJ) mice backcrossed onto the BALB/c versus C57BL/6 backgrounds. Our results confirm and further characterise the previously reported phenotype and identify strain-dependent differences in bone development, thymocyte development and myeloid hyperplasia and overall differences in morbidity and mortality.

## Results

### Targeted disruption of Ptpn2

The *Ptpn2* gene is located on chromosome 18 and has 7 exons spread over approximately 60 kb of genomic DNA. To generate a *Ptpn2* null mouse using Cre/LoxP targeting, a construct was designed with a PGK (Phosphoglycerate kinase)-Neo cassette flanked with FRT recombination sites inserted downstream of exon 2, and both exon 2 and PGK-neo flanked with LoxP recombination sites for deletion using Cre recombinase; excision of exon 2 would splice exon 1b to exon 3 to induce a frame-shift that would result in early termination (TAA) and a putative 87 residue protein (10,320 Da) incorporating 53 residues from the TCPTP N-terminus (**Fig. 1a**). The linearised construct was electroporated into C57BL/6 derived Bruce 4 embryonic stem (ES) cells. Correctly targeted Neo resistant clones were injected into blastocysts and high percentage chimeras crossed to C57BL/6J Oz-Cre deleter mice (Ozgene) to excise exon 2 and the Neo cassette (**Fig. 1b**) and thereon bred with C57BL/6J mice to eliminate the Cre recombinase (**Fig. 1c**). The resulting *Ptpn2* exon2-deleted mice (*Ptpn2^ex2+/ex2−^*) were maintained by breeding heterozygotes (**Fig. 1d**). *Ptpn2^ex2−/ex2−^* were born at the expected Mendelian frequency (**Table** S**1**). Western blot analyses using antibodies (6F3) to the TCPTP non-catalytic C-terminus confirmed that full length TCPTP protein was not detectable in any of the tissues examined in homozygous *Ptpn2^ex2−/ex2−^* mice (**Fig. 1e**). The hypothetical 87 residue peptide resulting from the splicing of exon 1b to 3 could not be detected with antibodies (6F7; raised against the first 38 residues of TCPTP) to the TCPTP N-terminus (**Fig. 1f**), consistent with the corresponding mRNA and/or protein being unstable and the mice being null for TCPTP.

### Morbidity and mortality in Ptpn2-deficient mice

**Figure 2:**
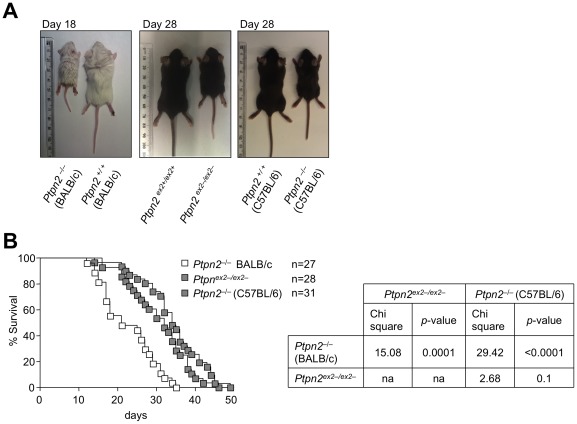
Runtiness and mortality in Ptpn2^−/−^ (BALB/c), Ptpn2^ex2−/ex2−^ and Ptpn2^−/−^ (C57BL/6) mice. (**a**) Images of representative 18 day-old *Ptpn2^−^* ^/*−*^ (BALB/c), 28 day-old *Ptpn2^ex2−/ex2−^* and 28 day-old *Ptpn2^−^* ^/*−*^ (C57BL/6) mice along with corresponding littermate wild type control mice. (**b**) Kaplan-Meier survival curves for *Ptpn2^−^* ^/*−*^ (BALB/c), *Ptpn2^ex2−/ex2−^* and *Ptpn2^−^* ^/*−*^ (C57BL/6) mice. Statistical analyses were performed using a Log Rank (Mantel-Cox) test with one degree of freedom.

At 2 weeks of age *Ptpn2^ex2−/ex2−^* (C57BL/6) mice were smaller than their *Ptpn2^ex2+/ex2−^* and wild type littermates, but otherwise appeared and behaved normally. As reported previously for *Ptpn2^−/−^* mice on the mixed BALB/c-129SJ background [25], backcrossed (eight generations) *Ptpn2^−/−^* (BALB/c) mice were runted and developed morbidity by 18–21 days of age, characterized by a hunched posture, piloerection, eyelid closure, decreased mobility and diarrhoea (**Fig. 2**; **Table S2**). The median survival for *Ptpn2^−/−^* (BALB/c) was 21 days (**Fig. 2b**). In contrast, the median survival for *Ptpn2^ex2−/ex2−^* mice was 32 days with some mice surviving for almost 50 days (**Fig. 2b**). Notably at 29 days of age *Ptpn2^ex2−/ex2−^* mice were runted, but behaved normally and did not exhibit the overt signs of morbidity evident in *Ptpn2^−/−^* (BALB/c) mice (**Table S2**). To determine if these phenotypic differences might be due to the respective BALB/c versus C57BL/6 backgrounds, we backcrossed the *Ptpn2^−/−^*(BALB/c) mice onto the C57BL/6/J background strain for seven generations. We found that the backcrossed *Ptpn2^−/−^* (C57BL/6/J) mice resembled *Ptpn2^ex2−/ex2−^* mice in appearance and behaviour with a median survival of 34 days (**Fig. 2**; **Table S2**; data not shown). These results indicate that *Ptpn2^ex2−/ex2−^* mice phenocopy *Ptpn2^−/−^* (C57BL/6) mice and that differences in morbidity and mortality between *Ptpn2^−/−^* (BALB/c) and *Ptpn2^ex2−/ex2−^* mice might be ascribed to differences in the background strain.

### Bone development in Ptpn2-deficient mice

**Figure 3:**
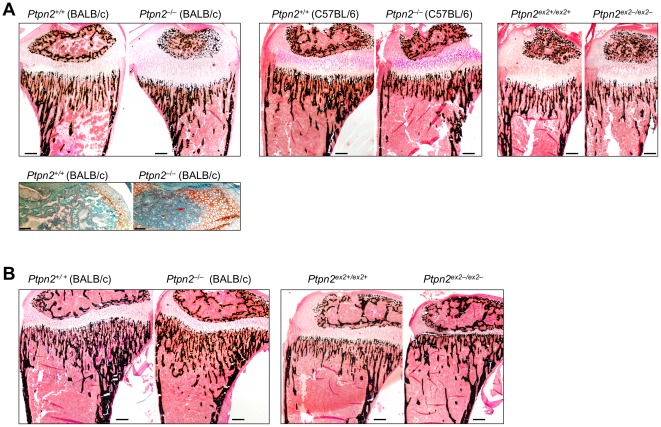
Bone development in Ptpn2^−/−^ (BALB/c) mice. Representative longitudinal sections of tibiae from (**a**) 14 day-old *Ptpn2^−^* ^/*−*^ (BALB/c), *Ptpn2^−^* ^/*−*^ (C57BL/6) and *Ptpn2^ex2−/ex2−^* mice, or (**b**) 21 day-old *Ptpn2^−^* ^/*−*^ (BALB/c) and *Ptpn2^ex2−/ex2−^* mice and their corresponding littermate wild type controls stained with the *von Kossa* technique (mineralized bone stained black). In a) safranin O staining [only shown for 14 day-old *Ptpn2^+^* ^/*+*^ (BALB/c) and *Ptpn2^−^* ^/*−*^ (BALB/c)] highlights the delayed cartilage (in orange) destruction; scale bar = 100 micron.

Previous studies have indicated that a bone marrow stromal cell deficiency underlies the inflammatory phenotype and overt morbidity in *Ptpn2^−/−^* (BALB/c-129SJ) mice [25]. More recent studies have shown that TCPTP deficiency in osteoblasts (C57BL/6J-129SJ) increases osteoclast activity *in vitro*, without significantly affecting the osteoblast or osteoclast number or bone formation/volume *in vivo* [31]. We characterized the bones of *Ptpn2^−/−^*(BALB/c) and *Ptpn2^ex2−/ex2−^* mice to determine whether the differences in morbidity might be associated with differences in bone development. Hind legs were isolated from 14 day-old *Ptpn2^−/−^* (BALB/c) and corresponding *Ptpn2^+/−^* and *Ptpn2^+/+^* littermates prior to the onset of overt morbidity [25], [26], as well as 14 day-old *Ptpn2^ex2−/ex2−^* and *Ptpn2^ex2+/ex2+^* littermate mice. Legs were fixed in 3.7% formaldehyde and processed for bone histology and histomorphometric analysis as described previously [32]. 14 day-old *Ptpn2^−/−^* (BALB/c) mice had significantly smaller skeletons, reflected in reduced femoral length and width, and delayed development of the secondary ossification centre, indicated by the high level of cartilage remaining in this zone (**Fig 3**). In addition, in the proximal tibial metaphysis, a region of bone remodeling, *Ptpn2^−/−^* (BALB/c) mice had increased trabecular bone volume, characterized by high trabecular number (TbN) and trabecular thickness (TbTh) and reduced trabecular separation (TbSp) (**Fig. 3**; **Table 1**). A lower level of remodeling was indicated by reduced osteoid deposition, and a significant reduction in osteoblast surface, along with a slight, but not statistically significant reduction in osteoclast surface (**Table 1**). Interestingly in 21 day-old *Ptpn2^−/−^* (BALB/c) mice, the delay in the secondary ossification centre was no longer evident and the increase in trabecular bone was less obvious (**Fig. 3**). In contrast to *Ptpn2^−/−^* (BALB/c), we found slightly, but not significantly lower femoral length in 14 day-old *Ptpn2^ex2−/ex2−^* mice, but no overt difference in femoral width or trabecular bone volume (**Fig. 3**; **Table 2**). Histological assessment indicated that *Ptpn2^−/−^* (C57BL/6) mice also did not exhibit any overt difference in size, nor increased trabecular bone at 14 days of age (**Fig. 3**). At 21 days of age *Ptpn2^ex2−/ex2−^* bones were overtly smaller, and this was associated with a striking reduction in the growth plate width; again there was no increase in trabecular bone (**Fig. 3**). Taken together, these results point towards TCPTP-deficiency resulting in strain-dependent differences in bone development and remodeling.

**Table 1:** Bone histomorphometry in Ptpn2^−/−^ (BALB/c) mice.

	*Ptpn2^+/+^* (n = 8)	*Ptpn2^+/−^* (n = 4)	*Ptpn2^−/−^* (n = 10)	*p* value *+/+* v/s *−/−*
**Femoral length (mm) ± SE**	9.5±0.3	9.2±0.2	7.8±0.2	0.002
**Femoral width (mm) ± SE**	0.93±0.01	0.92±0.04	0.83±0.01	0.002
**Trabecular bone volume BV/TV (%) ± SE**	4.8±0.6	4.2±0.1	8.9±1.0	0.006
**Trabecular number TbN (mm) ± SE**	2.4±0.2	2.3±0.4	3.9±0.4	0.006
**Trabecular thickness TbTh (µm) ± SE**	19.5±0.8	17.4±1.7	23.0±1.1	0.027
**Trabecular separation TbSp (µm) ± SE**	411±29	462±99	262±30	0.006
**Osteoid volume OV/BV (%) ± SE**	5.2±1.1	5.0±1.0	2.0±0.5	0.02
**Osteoid surface OS/BS (%) ± SE**	18.4±2.7	18.2±3.7	12.3±3.0	0.17
**Osteoid thickness (µm) ± SE**	2.6±0.2	2.4±0.1	1.9±0.1	0.004
**Osteoblast surface** **ObS/BS (%) ± SE**	29.7±3.9	27.6±6.1	17.6±4.0	0.04
**Osteoclast surface OcS/BS (%) ± SE**	21.4±2.5	28.3±6.6	15.3±2. 5	0.08
**Number of Osteblasts/Bone perimeter Nob/BPm (/mm) ± SE**	18.7±2.0	11.0±4.0	12.0±2.6	0.09
**Number of Osteclasts/Bone perimeter Noc/BPm (/mm) ± SE**	5.6±0.8	8.5±2. 6	4.1±0.6	0.10

Bone volume/Trabecular bone volume (BV/TV), trabecular number (TbN), trabecular thickness (TbTh) and trabecular separation (TbSp) in 14 day-old *Ptpn2^+/+^* (BALB/c), *Ptpn2^+/−^* (BALB/c) and *Ptpn2^−/−^* (BALB/c) mice. Osteoid surface/bone surface (OS/BS), osteoid thickness, osteoid volume/bone volume (OV/BV), osteoblast surface/bone surface (ObS/BS), osteoclast surface/bone surface (OcS/BS), number of osteoblasts/bone perimeter (Nob/BPm) and number of osteoclasts/bone perimeter (Noc/BPm) are also shown. Results shown are means ± SEM; *p* values are for *Ptpn2^+/+^* versus *Ptpn2^−/−^* mice and were determined using a two-tailed Mann-Whitney U Test.

**Table 2:** Bone histomorphometry in Ptpn2^ex2−/ex2−^ (C57BL/6) mice.

	*Ptpn2^ex2+/ex2+^* (n = 7–8)	*Ptpn2^ex2−/ex2−^* (n = 7–8)	*p* value *+/+* v/s *−/−*
**Femoral length (mm) ± SE**	8.6±0.1	8.0±0.3	0.097
**Femoral width (mm) ± SE**	1.04±0.03	1.04±0.03	1.000
**Trabecular bone volume BV/TV (%) ± SE**	2.95±0.70	3.68±0.41	0.382
**Trabecular number TbN (mm) ± SE**	1.46±0.32	1.67±0.19	0.645
**Trabecular thickness TbTh (µm) ± SE**	20.0±0.8	22.7±1.7	0.130
**Trabecular separation TbSp (µm) ± SE**	1253±495	646.9±93	0.645

Bone volume/Trabecular bone volume (BV/TV), trabecular number (TbN), trabecular thickness (TbTh) and trabecular separation (TbSp) in 14 day-old *Ptpn2^ex2+/ex2+^* (C57BL/6) and *Ptpn2^ex2−/ex2−^* (C57BL/6) mice. Results shown are means ± SEM; *p* values are for *Ptpn2^+/+^* versus *Ptpn2^−/−^* mice and were determined using a two-tailed Mann-Whitney U Test.

### Body composition in Ptpn2-deficient mice

Given the growth retardation, but discordant morbidity in *Ptpn2^−/−^* (BALB/c) versus *Ptpn2^−/−^* (C57BL/6) and *Ptpn2^ex2−/ex2−^* mice, we assessed the impact of TCPTP deficiency on body composition by dual energy X-ray absorptiometry (DEXA) at 18 and 28 days as appropriate (**Tables 3** **–** **4**). Lean mass, fat mass and bone mineral content and density were assessed in *Ptpn2^ex2−/ex2−^* and *Ptpn2^−/−^* (C57BL/6) mice and compared to that of *Ptpn2^−/−^*(BALB/c) mice and their corresponding heterozygous and/or wild type littermates. Lean mass, fat mass and bone mineral content but not bone mineral density, were reduced in *Ptpn2^ex2−/ex2−^*, *Ptpn2^−/−^* (C57BL/6) and *Ptpn2^−/−^*(BALB/c) mice when compared to their corresponding controls, consistent with the overt growth retardation (**Tables 3** **–** **4**). However, lean masses normalized to total body weight were unaltered in *Ptpn2^ex2−/ex2−^*, *Ptpn2^−/−^* (C57BL/6) and *Ptpn2^−/−^*(BALB/c) mice when compared to controls, in line with the mice being proportionately smaller and the difference in growth being developmental in nature. Relative fat masses were decreased in *Ptpn2^ex2−/ex2−^*, *Ptpn2^−/−^* (C57BL/6) and *Ptpn2^−/−^*(BALB/c) mice when compared to controls, consistent with TCPTP deficiency affecting energy balance, whereas bone mineral densities normalized to body weight were increased (**Tables 3** **–** **4**); at 18 days of age the increase in bone mineral density was greatest in *Ptpn2^−/−^* (BALB/c) mice consistent with the strain-dependent differences in bone development as revealed by histology and histomorphometry (**Fig. 3** **;** **Table 1**). Overall there were no overt differences in body composition between *Ptpn2^ex2−/ex2−^*, *Ptpn2^−/−^* (C57BL/6) and *Ptpn2^−/−^*(BALB/c) mice, consistent with the strain-dependent differences in morbidity and mortality being independent of gross body composition.

**Table 3:** Body composition in 18 day-old Ptpn2^−/−^ (BALB/c), Ptpn2^ex2−/ex2−^ and Ptpn2^−/−^ (C57BL/6) mice.

	*Ptpn2* (BALB/c)			*Ptpn2^ex2/ex2^*			*Ptpn2* (C57BL/6)		
Age 18 d	+/+ (n = 13)	*−*/*−* (n = 5)	*p*-value	+/+ (n = 10)	*−*/*−* (n = 8)	*p*-value	+/+ (n = 9)	*−*/*−* (n = 8)	*p*-value
**Lean Mass (g) ±SE**	8.7±0.4	4.7±0.6	0.002	6.8±0.3	5.1±0.6	0.03	9.4±0.8	6.0±0.6	0.01
**Lean Mass/Body Weight (%) ±SE**	74.0±1.1	84.0±2.4	0.003	77.0±0.5	81.0±1.8	0.13	80.0±0.7	82.0±3.8	0.69
**Fat Mass (g) ±SE**	3.2±0.3	0.9±0.1	0.002	2.1±0.1	1.2±0.2	0.01	2.4±0.2	1.3±0.3	0.01
**Fat Mass/Body Weight (%) ±SE**	26.2±1.1	16.5±2.3	0.006	23.2±0.6	18.5±1.9	0.16	19.9±0.7	18.3±3.8	0.69
**BMC (g) ±SE**	0.07±0.01	0.03±0.01	0.06	0.04±0.01	0.03±0.01	0.42	0.10±0.02	0.05±0.01	0.04
**BMC/Body Weight (%) ±SE**	0.54±0.09	0.53±0.21	1.00	0.42±0.05	0.48±0.06	0.41	0.80±0.09	0.60±0.11	1.00
**BMD ±SE [BMC(g)/Area (cm^2^)]**	0.030±0.001	0.026±0.002	0.18	0.030±0.001	0.025±0.001	0.67	0.030±0.001	0.027±0.001	0.09
**BMD/Body Weight (%)**	0.25±0.01	0.50±0.09	0.006	0.29±0.01	0.42±0.04	0.0004	0.27±0.02	0.40±0.04	0.01

Body composition [lean, fat, bone mineral content (BMC) and bone mineral density (BMD)] in *Ptpn2^−^* ^/*−*^ (BALB/c), *Ptpn2^ex2−/ex2−^* (C57BL/6) and *Ptpn2^−^* ^/*−*^ (C57BL/6) mice and in the corresponding wild type littermates measured by DEXA and normalised to total body weight. Results shown are means ± SEM; *p* values are for *Ptpn2^+/+^* versus *Ptpn2^−/−^* mice and were determined using a two-tailed Mann-Whitney U Test.

**Table 4:** Body composition in 28 day-old Ptpn2^ex2−/ex2−^ and Ptpn2^−/−^ (C57BL/6) mice.

	*Ptpn2^ex2/ex2^*			*Ptpn2* (C57BL/6)		
	+/+ (n = 10)	*−*/*−* (n = 8)	*p* value	+/+ (n = 9)	*−*/*−* (n = 8)	*p* value
**Lean Mass (g) ±SE**	13.8±0.7	5.7±0.5	0.0001	14.7±1.0	6.6±0.5	0.0003
**Lean Mass/Body Weight (%) ±SE**	81.0±0.7	86.0±0.8	0.0004	85.0±0.7	87.0±1.0	0.09
**Fat Mass (g) ±SE**	3.3±0.2	0.9±0.1	0.0001	2.7±0.3	0.9±0.1	0.0003
**Fat Mass/Body Weight (%) ±SE**	19.3±0.7	13.4±0.7	0.0001	15.2±0.7	13.1±1.2	0.18
**BMC (g) ±SE**	0.19±0.01	0.05±0.01	0.0001	0.23±0.02	0.08±0.01	0.0006
**BMC/Body Weight (%) ±SE**	1.09±0.03	0.70±0.13	0.06	1.29±0.07	1.02±0.13	0.09
**BMD ±SE [BMC(g)/Area (cm^2^)]**	0.037±0.001	0.025±0.001	0.0001	0.041±0.002	0.028±0.001	0.0006
**BMD/Body Weight (%)**	0.22±0.01	0.40±0.04	0.0001	0.24±0.01	0.38±0.01	0.0006

Body composition [lean, fat, bone mineral content (BMC) and bone mineral density (BMD)] in 28 day-old *Ptpn2^ex2−/ex2−^* (C57BL/6) and *Ptpn2^−^* ^/*−*^ (C57BL/6) mice and in the corresponding wild type littermates measured by DEXA and normalised to total body weight. Results shown are means ± SEM; *p* values are for *Ptpn2^+/+^* versus *Ptpn2^−/−^* mice and were determined using a two-tailed Mann-Whitney U Test.

### T cell development in Ptpn2-deficient mice

**Figure 4:**
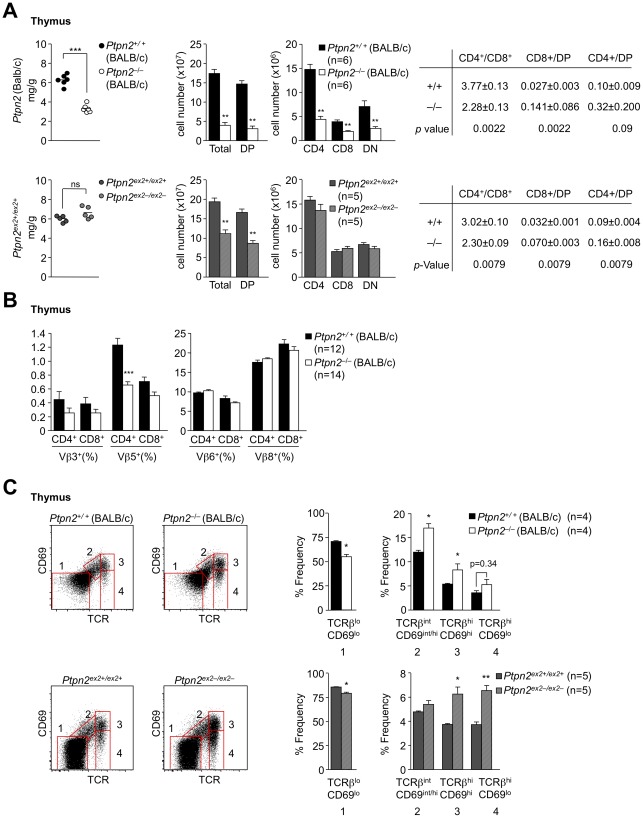
Thymocyte development in 18 day-old Ptpn2^−/−^ (BALB/c) and Ptpn2^ex2−/ex2−^ mice. (**a**) Thymi from the indicated 18 day-old littermates were weighed using an analytical balance and normalised to body weight. Thymocytes were stained with fluorochrome-conjugated antibodies against CD4 and CD8 and analyzed by flow cytometry. Cells were gated for the DP, SP and DN stages and absolute numbers and the indicated ratios determined. (**b**) Thymocytes from *Ptpn2^+/+^* and *Ptpn2^−^* ^/*−*^ (BALB/c) littermates were stained with fluorochrome-conjugated antibodies against CD4, CD8, TCR-Vβ3, -5, -6 and -8 and analyzed by flow cytometry. Cells were gated for the CD4 and CD8 SP stages and the percentages of TCR-Vβ3, -5, -6 and -8 T cells determined. (**c**) Thymocytes from the indicated 18 day-old mice were stained with fluorochrome-conjugated antibodies against CD4, CD8, TCRβ and CD69 and analyzed by flow cytometry. Cells were gated for the different developmental stages according to the expression of the positive selection markers TCRβ and CD69. Results in (**a–c**) are means ± SEM for the indicated number of mice and are representative of at least two independent experiments; significance determined using a two-tailed Mann-Whitney U Test; *p<0.05 ** p<0.01 *** p<0.001.

**Figure 5:**
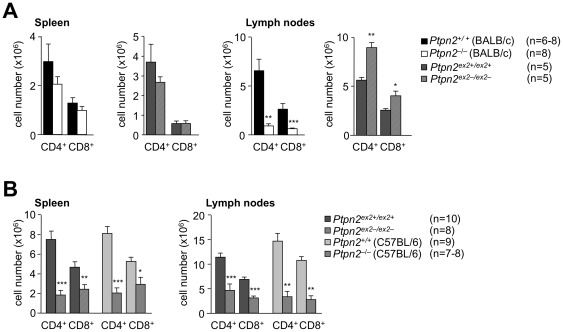
Peripheral T cells in Ptpn2-deficient mice. (**a**) Splenocytes or peripheral lymph node cells from 18 day-old *Ptpn2^−^* ^/*−*^ (BALB/c) mice, 18 day-old *Ptpn2^ex2−/ex2−^* mice and their corresponding littermates were stained with fluorochrome-conjugated antibodies against CD4, CD8 and TCRβ and analysed by flow cytometry and absolute numbers determined. (**b**) Splenocytes or peripheral lymph node cells from 28 day-old *Ptpn2^ex2−/ex2−^* mice, 29 day-old *Ptpn2^−/−^* (C57BL/6) mice and their corresponding littermates were stained with fluorochrome-conjugated antibodies against CD4, CD8 and TCRβ and analysed by flow cytometry and absolute numbers determined. Results shown in (**a–b**) are means ± SEM for the indicated number of mice and are representative of at least two independent experiments; significance determined using a two-tailed Mann-Whitney U Test; *p<0.05 ** p<0.01 *** p<0.001.

**Figure 6:**
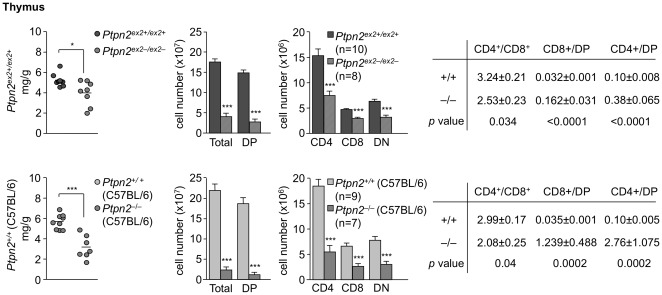
Thymoycte development in 28 day-old Ptpn2^ex2−/ex2−^ and Ptpn2^−/−^ (C57BL/6) mice. Thymi from the indicated littermate mice were weighed using an analytical balance and normalised to body weight. Thymocytes were stained with fluorochrome-conjugated antibodies against CD4 and CD8 and analyzed by flow cytometry. Cells were gated for the DP, SP and DN stages and absolute numbers and the indicated ratios determined. Results shown are means ± SEM for the indicated number of mice and are representative of at least two independent experiments; significance determined using a two-tailed Mann-Whitney U Test; *p<0.05 *** p<0.001.

T cell progenitor cells arise in the bone marrow and enter the thymus to form double negative (DN) cells that lack surface CD4 and CD8 and undergo maturation to develop into double positive (DP) thymocytes (CD4^+^CD8^+^) that express CD4 and CD8. DP thymocytes that are positively and negatively selected form single positive (SP) thymocytes [CD4^+^CD8^−^, CD4^−^CD8^+^] that ultimately leave the thymus as CD4^+^ and CD8^+^ naïve T cells [33]. We compared thymocyte development in 18 day-old *Ptpn2^−/−^* (BALB/c) versus *Ptpn2^ex2−/ex2−^* mice and their corresponding littermates (**Fig. 4** **)**. 18 day-old *Ptpn2^−/−^* (BALB/c) mice exhibited overt thymic atrophy associated with a 4–5 fold decrease in total cellularity; DN, DP and SP thymocytes (**Fig. 4a**), in particular in the CD4^+^CD8^−^ lineage, were decreased as reported previously for *Ptpn2^−/−^* (BALB/c-129SJ) mice [25]. In contrast thymic atrophy was not evident in *Ptpn2^ex2−/ex2−^* mice and total and DP thymocytes were decreased by only 50%, whereas SP thymocytes remained unaltered; thymic atrophy was also not evident in 18 day-old *Ptpn2^−/−^* (C57BL/6) mice (data not shown). Interestingly, SP/DP ratios (in particular CD4^−^8^+^/DP ratios) were significantly increased in both *Ptpn2^−/−^* (BALB/c) and *Ptpn2^ex2−/ex2−^* 18 day-old mice (**Fig. 4a**). The enhanced SP/DP ratios suggested that TCPTP-deficiency altered thymocyte development. One possibility is that thymocyte negative selection might be defective in TCPTP-deficient mice and thus contribute to enhanced SP generation. However, negative selection as monitored by the deletion of Vβ5+ and Vβ3+ TCR SPs by the endogenous MMTV (mouse mammary tumour provirus) 6 and 8 superantigens in 14–16 day-old *Ptpn2^−/−^* (BALB/c) mice was not diminished, but rather modestly enhanced (**Fig. 4b**). Another possibility is that positive selection might be enhanced. Thymocyte positive selection can be minimally subdivided into four progressive stages based on changes in the expression of TCRβ and CD69 [34]. Pre-selection DP cells are defined as TCRβ^lo^CD69^lo^ (stage 1), DPs initiating positive selection are TCRβ^int^CD69^int/hi^ (stage 2), whereas thymocytes in the process of positive selection are TCRβ^hi^CD69^hi^ (stage 3) and SPs that have completed positive selection are TCRβ^hi^CD69^lo^ (stage 4) [34]. We noted increases in the number of *Ptpn2^−/−^* (BALB/c) thymocytes initiating, undergoing and completing positive selection (**Fig. 4c**). Even greater increases in thymocytes initiating and completing positive selection were noted in 18 day-old *Ptpn2^ex2−/ex2−^* mice (**Fig. 4c**). Taken together, these results are consistent with TCPTP deficiency enhancing both negative and positive selection. Nonetheless, in keeping with the effects on thymic cellularity, we found that peripheral lymph node CD4+ and CD8+ T cells were reduced in *Ptpn2^−/−^* (BALB/c) mice and increased in *Ptpn2^ex2−/ex2−^* mice (**Fig. 5**). Given the differences in thymic atrophy/cellularity in 18 day-old *Ptpn2^−/−^* (BALB/c) versus *Ptpn2^ex2−/ex2−^* mice, we also monitored thymocyte subsets in 28–29 day-old *Ptpn2^−/−^* (C57BL/6) and *Ptpn2^ex2−/ex2−^* mice. Thymic atrophy was evident in both 28 day-old *Ptpn2^−/−^* (C57BL/6) and 29 day-old *Ptpn2^ex2−/ex2−^* mice and coincided with profound decreases in total, DN, DP and SP thymic cellularity, as seen in 18 day *Ptpn2^−/−^* (BALB/c) mice (**Fig. 6**). These results point towards both age- and strain-dependent differences in thymic atrophy versus thymocyte development and suggest that the overt thymic atrophy may be a consequence of extrinsic influences.

### Splenomegaly and lymphadenopathy in Ptpn2-deficient mice

**Figure 7:**
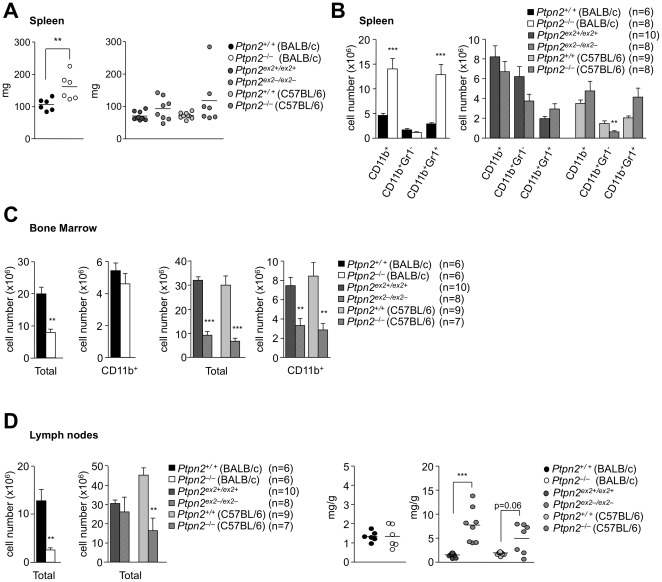
Splenomegaly, myeloid development and lymphadenopathy in Ptpn2-deficient mice. (**a**) Spleen weights from 18 day-old *Ptpn2^−^* ^/*−*^ (BALB/c) and 28 day-old *Ptpn2^ex2−/ex2−^* and *Ptpn2^−^* ^/*−*^ (C57BL/6) mice and corresponding wild type littermate mice. (**b**) Splenocytes or (**c**) bone marrow (tibia and femur) cells were stained with fluorochrome-conjugated antibodies to CD11b and Gr1 and analysed by flow cytometry; absolute numbers of CD11b^+^, CD11b^+^Gr1^+^ and CD11b^+^Gr1^−^ were determined (**d**) Weights of pooled peripheral lymph nodes from 18 day-old *Ptpn2^−^* ^/*−*^ (BALB/c) and 28 day-old *Ptpn2^ex2−/ex2−^* and *Ptpn2^−^* ^/*−*^ (C57BL/6) mice and corresponding wild type littermate mice. Results shown in (**a–d**) are means ± SEM for the indicated number of mice and are representative of at least two independent experiments; significance determined using a two-tailed Mann-Whitney U Test; *p<0.05 ** p<0.01 *** p<0.001.

In addition to thymic atrophy *Ptpn2^−/−^* (BALB/c-129SJ) mice develop splenomegaly after two weeks of age [25]. Splenomegaly was evident in 18 day-old *Ptpn2^−/−^*(BALB/c) mice (**Fig. 7a**), albeit not as pronounced as that reported previously for *Ptpn2^−/−^* (BALB/c-129SJ) mice [25]. In contrast, spleen weights were not significantly altered in 28 day-old *Ptpn2^−/−^* (C57BL/6) or *Ptpn2^ex2−/ex2−^* mice (**Fig. 7a**). Splenomegaly in *Ptpn2^−/−^* (BALB/c-129SJ) mice has been associated with an increase in the red pulp and an accumulation of myeloid cells [25]. We found that total CD11b^+^ and CD11b^+^Gr^+^ (but not CD11b^+^Gr*^−^*) myeloid cells were increased dramatically in the spleens of *Ptpn2^−/−^* (BALB/c) mice, but not in *Ptpn2^−/−^* (C57BL/6), or *Ptpn2^ex2−/ex2−^* (C57BL/6) mice when compared to their corresponding littermates (**Fig. 7b**). On the other hand CD11b^+^ cells were decreased in the bone marrow of *Ptpn2^−/−^* (C57BL/6) and *Ptpn2^ex2−/ex2−^* mice, consistent with a defect in myeloid development, however this was not evident in *Ptpn2^−/−^* (BALB/c) mice (**Fig. 7c**). *Ptpn2^−/−^* (BALB/c), *Ptpn2^−/−^* (C57BL/6) and *Ptpn2^ex2−/ex2−^* mice had decreased bone marrow cellularity (**Fig. 7c**), in keeping with their smaller bones and increased relative bone mineral density (**Tables 1** **–** **2**).

Lymphadenopathy and increases in lymph node (LN) cellularities have also been reported for *Ptpn2^−/−^* (BALB/c-129SJ) mice [25]. Lymphodenopathy was not evident in *Ptpn2^−/−^* (BALB/c) mice (**Fig. 7d**), rather, LN weights (normalised to total body weight) were unaltered and cellularity reduced (∼5 fold) in *Ptpn2^−/−^* (BALB/c) mice. On the other hand, LN weights were modestly increased in *Ptpn2^ex2−/ex2−^* mice and LN weights trended higher in *Ptpn2^−/−^* (C57BL/6) mice, whereas cellularity (4–15 µm; Z2 coulter counter) was unaltered in *Ptpn2^ex2−/ex2−^* mice and decreased (∼2.9 fold) in *Ptpn2^−/−^* (C57BL/6) mice (**Fig. 7d**). These results indicate that the effects of TCPTP deficiency on splenomegaly, myeloid development and lymphodenopathy are strain-dependent.

### B cell development in Ptpn2-deficient mice

**Figure 8:**
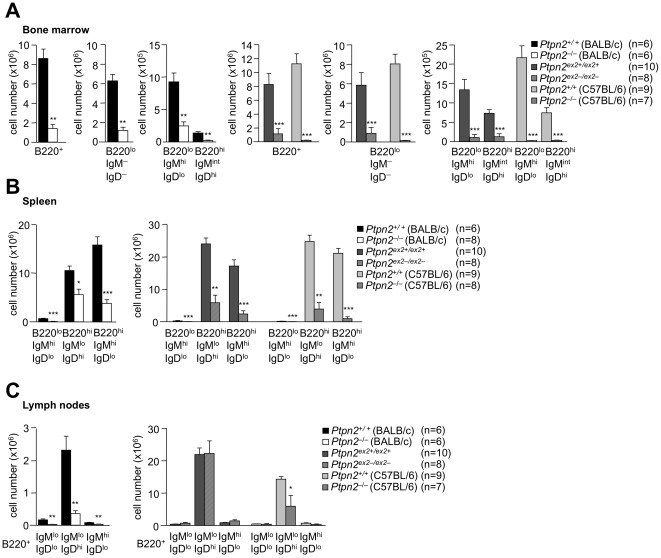
B cell development in Ptpn2-deficient mice. Bone marrow cells (pooled from one tibia and one femur), splenocytes or pooled peripheral lymph node cells from 18 day-old *Ptpn2^−^* ^/*−*^ (BALB/c), 28 day-old *Ptpn2^ex2−/ex2−^* and 29 day-old *Ptpn2^−^* ^/*−*^ (C57BL/6) mice and their corresponding littermates (**a–c**) were stained with fluorochrome-conjugated antibodies to CD45R(B220), IgM and IgD and analysed by flow cytometry. Absolute numbers of progenitor (B220^lo^IgM^lo^IgD^lo^), immature (B220^lo^IgM^hi^ IgD^lo^), mature (B220^hi^IgM^int^IgD^hi^) B cells and B1 (B220^lo^ IgM^hi^IgD^lo^), follicular (B220^hi^IgM^lo^IgD^hi^) and marginal zone (B220^hi^IgM^hi^IgD^lo^) B cells were determined. Results shown are means ± SEM for the indicated number; significance determined using a two-tailed Mann-Whitney U Test; *p<0.05 ** p<0.01 *** p<0.001.

We next assessed B cell development in 18 day-old *Ptpn2^−/−^* (BALB/c), 28 day-old *Ptpn2^−/−^* (C57BL/6) and 29 day-old *Ptpn2^ex2−/ex2−^* mice (versus corresponding littermates). B cells originate from precursors in the bone marrow and undergo sequential maturation and V(D)J recombination developing into immature B cells that express B cell receptor and membrane-bound IgM [35]. Immature B cells emigrate to the spleen where they mature further into three broad populations according to anatomical localization, phenotypic characterization and function: follicular B cells (IgM^lo^IgD^hi^B220^hi^), marginal zone B cells (IgM^hi^IgD^lo^ B220^hi^) and B-1 cells (IgM^hi^IgD^lo^ B220^lo^) [35]. Previous studies have shown that B cell intrinsic defects and bone marrow stromal abnormalities in TCPTP-deficient mice lead to an early block in B cell development [25], [28]. Consistent with this we found a dramatic reduction in total B220^+^ B cells, IgM^−^IgD^−^B220^lo^ progenitor B cells, IgM^hi^IgD^lo^B220^lo^ immature B cells and IgM^int^IgD^hi^B220^hi^ mature recirculating B cells in bone marrow and decreased splenic marginal zone B cells, follicular B cells and B-1 cells (**Fig. 8a–b**). Although bone marrow B cell development was perturbed in all three mouse lines, the impact of TCPTP deficiency on the development of splenic B cells was most dramatic in *Ptpn2^−/−^* (C57BL/6) and *Ptpn2^ex2−/ex2−^* mice (**Fig. 8b**). Conversely, LN B cell subsets were most dramatically altered in *Ptpn2^−/−^* (BALB/c), with no overt differences seen in *Ptpn2^ex2−/ex2−^* mice (**Fig. 8c**).

### Erythropoiesis in Ptpn2-deficient mice

**Figure 9:**
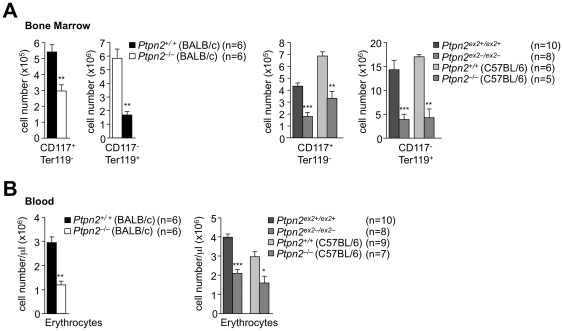
Erythrocyte development in Ptpn2-deficient mice. (**a**) Bone marrow cells (pooled from one tibia and one femur) from 18 day-old *Ptpn2^−^* ^/*−*^ (BALB/c) and 28 day-old *Ptpn2^ex2−/ex2−^* and *Ptpn2^−^* ^/*−*^ (C57BL/6) mice and corresponding littermate wild type control mice were stained with fluorochrome-conjugated antibodies to CD117 and Ter119 and analysed by flow cytometry. Absolute numbers of progenitor (CD117^+^ Ter119^−^) and erythroid (CD117^−^Ter119^+^) cells were determined. (**b**) Red blood cells were quantified (identified in the forward and side scatter according to size and granularity) by flow cytometry. Results shown are means ± SEM for the indicated number of mice and are representative of two independent experiments; significance determined using a two-tailed Mann-Whitney U Test; *p<0.05 ** p<0.01 *** p<0.001.

*Ptpn2^−/−^* (BALB/c-129SJ) mice develop severe anemia by 21 days of age associated with defective bone marrow erythropoiesis [25]. Erythropoiesis is a dynamic process that involves multiple differentiation steps and progression from early progenitor cells to enucleated red blood cells. Pluripotent hematopoietic stem cells and multipotent progenitor cells in the bone marrow give rise to committed erythroid precursors, which sequentially develop into mature erythrocytes [36]. We found that CD117^+^Ter119^−^ progenitor cells and CD117^−^Ter119^+^ erythroid cells (early pro-erythroblast to mature erythrocyte) were decreased by more than 80% in *Ptpn2^−/−^* (BALB/c), *Ptpn2^−/−^* (C57BL/6) and *Ptpn2^ex2−/ex2−^* mice when compared to their corresponding littermates (**Fig. 9a**). Circulating erythrocytes were also reduced, but this was most dramatic in *Ptpn2^−/−^* (BALB/c) mice (**Fig. 9b**). Interestingly, the decrease in erythroid cells was greater than the decrease in erythrocytes (**Fig. 9**), consistent with tissues such as the spleen and liver compensating for the defective bone marrow erythropoiesis. These results indicate that erythroid development is defective in TCPTP-deficient mice.

## Discussion

In this study we have generated mice that are globally deficient for the tyrosine phosphatase TCPTP using Cre/LoxP recombination to remove both *Ptpn2* exon 2 and the Neomycin resistance cassette used for ES cell selection. The phenotype of these mice reaffirms that reported previously for mice generated by homologous recombination, where the Neo cassette was left in place [25]. Thus, our studies indicate that the morbidity and mortality and the perturbations in hematopoiesis and erythropoiesis in *Ptpn2^−/−^* (BALB/c) can be ascribed to a deficiency in TCPTP, rather than extrinsic influences that may arise from the presence of the Neo resistance gene. Furthermore, our studies define strain-dependent differences in morbidity and mortality and differences in thymocyte, myeloid and bone development.

Global TCPTP-deficiency was associated with differences in morbidity and mortality on the BALB/c versus C57BL/6 background strains. The outward signs of morbidity in *Ptpn2^−/−^* (BALB/c) mice, including hunched posture, piloerection, decreased mobility, eyelid closure and diarrhoea were not evident in the corresponding mice on the C57BL/6 background. Moreover, lifespan was prolonged in TCPTP-deficient on the C57BL/6 background. The underlying reason(s) for the strain-dependent differences in morbidity and mortality is/are not clear. One possible cause may be differences in the myeloid compartment. Although growth retardation and defects in lymphocyte and erythroid development were evident irrespective of the background strain, the impact on the myeloid compartment was distinct. Despite bone marrow cellularity being decreased in both 28 day-old C57BL/6 and 18 day-old BALB/c strain TCPTP-deficient mice (in line with their smaller size), only C57BL/6 TCPTP-deficient mice exhibited a corresponding decrease in bone marrow CD11b^+^ cells. Thus, monocytic/granulocytic development may be increased in *Ptpn2^−/−^* (BALB/c) mice. This was accompanied by a dramatic increase in CD11b^+^Gr1^+^, but not CD11b^+^Gr1^−^ cells in the spleens of *Ptpn2^−/−^* (BALB/c) mice. The anti-granulocyte receptor 1 (Gr1) antibody RB6-8C5 binds to Ly6G on the surface of granulocytes and Ly6C present on both granulocytes and monocytes/macrophages and some lymphocytes. Two major monocytic subsets exist in the periphery, the ‘inflammatory’ and ‘circulating’ monocytes [37]. Inflammatory monocytes express Ly6C (Gr1^+^) and migrate to the spleen and inflamed tissues, where they become macrophages, whereas circulating monocytes are Gr1^−^ and serve to replenish resident tissue macrophages. The dramatic increase in granulocytes and/or inflammatory macrophages in the spleens of *Ptpn2^−/−^* (BALB/c) mice is consistent with inflammation and previous studies establishing the development of progressive systemic inflammatory disease in *Ptpn2^−/−^* (BALB/c-129SJ) mice [25].

Our studies also define strain-dependent differences in thymic atrophy and thymocyte development that might contribute to the differences in morbidity in BALB/c versus C57BL/6 strain TCPTP-deficient mice. In particular, thymic atrophy and the overt decrease in thymic cellularity were evident sooner in the *Ptpn2^−/−^* (BALB/c) mice. The reasons for the strain-dependent temporal differences in thymic atrophy are not clear. The decrease in thymocyte cellularity in BALB/c and C57BL/6 strain TCPTP-deficient mice might be attributable at least in part to the non-specific depletion of DP thymocytes by inflammatory cytokines such as TNF and IFNγ that are increased in *Ptpn2^−/−^* (BALB/c-129SJ) mice [26] and have been linked previously with the induction of DP apoptosis *in vivo* [38]. However, this might also be a consequence of defective lymphoid progenitor development in the bone marrow. In future studies it will be important to determine whether there are strain-dependent differences in inflammation and lymphoid progenitor development in *Ptpn2^−/−^* mice. Nevertheless, despite the decreased cellularity, we found that TCPTP-deficiency enhanced both positive and negative selection and overall resulted in increased SP/DP ratios. The enhanced positive selection was evident in particular in young *Ptpn2^ex2−/ex2−^* and reflected by an increase in peripheral T cell numbers. These results are consistent with our recent analyses of thymocyte/T cell-specific TCPTP-deficient (*Lck*-Cre;*Ptpn2^lox/lox^*) mice, where positive selection and SP/DP ratios are also increased [39]. Although T cell-specific TCPTP deficiency results in inflammation and autoimmunity in aged mice [24], it is possible that the enhanced T cell numbers in young *Ptpn2^ex2−/ex2−^* might be beneficial.

Phenotypic differences in different strains of mice carrying targeted null mutations have been reported previously and linked with the existence of second-site modifier genes that affect penetrance [40], [41], [42], [43], [44]. In particular, mice lacking the retinoblastoma (Rb)-related *p107* gene develop growth deficiency on the BALB/c, but not C57BL/6 background strains [40]. Indeed, the overall phenotype of *p107^−/−^* (BALB/c) mice is highly reminiscent of that seen in *Ptpn2^−/−^* (BALB/c) mice. In addition to postnatal growth retardation, *p107^−/−^* (BALB/c) mice have a higher incidence of morbidity and exhibit myeloid hyperplasia [40] and a suppression of B-lymphopoiesis and erythropoiesis [45]. The myeloid hyperplasia in p130-deficient mice is linked with perturbations in the bone marrow microenvironment [45]. *Ptpn2^−/−^* (BALB/c-129SJ) mice also exhibit bone marrow stromal cell defects associated with increased INFγ production [26], [28]. The defect(s) in the bone marrow microenvironment in *Ptpn2^−/−^* (BALB/c-129SJ) mice contribute(s) to morbidity and mortality and has/have been shown to be responsible for the block in B cell development [25], [28]. Although we did not assess if TCPTP-deficiency results in strain-dependent differences in the bone marrow stroma, we did observe a striking difference in bone development in *Ptpn2^−/−^* (BALB/c) versus *Ptpn2^ex2−/ex2−^* mice.

At 14 days of age *Ptpn2^−/−^* (BALB/c) mice had smaller skeletons due to a delay in bone development, as reflected by the large volume of unresorbed cartilage in the secondary ossification centre (at the epiphysis). Moreover, we found that trabecular bone was increased significantly in *Ptpn2^−/−^* (BALB/c) mice, comprising mainly of woven bone rather than more mature lamellar bone, consistent with the developmental delay. Our findings are consistent with global TCPTP deficiency delaying bone development and decreasing bone turnover on the BALB/c background. Interestingly, in 21 day-old *Ptpn2^−/−^* (BALB/c) mice the delay in the secondary ossification was no longer evident and only modest effects on trabecular bone were seen. Therefore, these results are not inconsistent with a recent study that has reported that 21 day-old *Ptpn2^−/−^* (BALB/c) mice exhibit spontaneous synovitis associated with synovial inflammation and increased osteoclast density and decreased rather than increased bone volume [46]. The overt differences we observed in bone development in *Ptpn2^−/−^* (BALB/c) mice were not present in *Ptpn2^ex2−/ex2−^* mice at 14 days of age. Moreover in contrast to 21 day-old *Ptpn2^−/−^* (BALB/c) mice, TCPTP deficiency on the C57BL/6 background resulted in a striking narrowing of the growth plate at 21 days of age; an influence that would cause a slowing of longitudinal growth and might therefore be responsible for the smaller skeletons in these mice. Therefore, although TCPTP-deficiency results in smaller skeletons irrespective of strain by 21 days of age, the underlying causes might be different and strain-dependent. A similar growth plate phenotype to that of the *Ptpn2^ex2−/ex2−^* mice has been noted previously in growth hormone (GH) receptor deficient mice [47]. Interestingly, mice that lack TCPTP specifically in neuronal cells (*Nes*-Cre;*Ptpn2^lox/lox^*) are also runted and this is associated with decreased circulating GH, which, amongst other influences, affects post-natal growth [39].

It is well established that the bone microenvironment plays a central role in hematopoietic stem cell fate [48], [49]. Further studies should focus on delineating the precise cellular and molecular mechanisms that underlie the strain-dependent differences in bone development and potentially bone marrow stroma in *Ptpn2^−/−^* mice and the relative contributions of bone to the perturbations in hematopoiesis and erythropoiesis and the morbidity and mortality that are associated with global TCPTP-deficiency.

## Methods

### Ethics statement

All experiments were performed in accordance with the NHMRC Australian Code of Practice for the Care and Use of Animals and approved by the Monash University School of Biomedical Sciences Animal Ethics Committee (Ethics number SOBSB/B/2008/53).

### Materials

Mouse anti-actin from Thermo Scientific (Fremont, CA), anti-tubulin (Ab-5) from Sigma-Aldrich (St Louis, MO) and anti-TCPTP (6F3, 6F7) from Medimabs (Quebec, Canada). The following fluorochrome-conjugated antibodies for flow cytometry from BD Biosciences (San Jose, CA) were used for staining: fluorescein isothiocyanate (FITC)-conjugated anti-mouse CD45R(B220) (RA3-6B2), FITC-conjugated anti-TCR-Vβ3 (KJ25), FITC-conjugated anti-TCR-Vβ5.1/5.2 (MR9-4), FITC-conjugated anti-TCR-Vβ6 (RR4-7), FITC-conjugated anti-TCR-Vβ8.1-8.2 (MR5-2), FITC-conjugated anti-TCRβ (H57-597), FITC-conjugated anti-CD117 (2B8), phycoerythrin (PE)-cyanine 7-conjugated anti-CD4 (RM4-5), PE-cyanine dye7-conjugated anti-CD69 (H1.2F3), PE-cyanine dye7-conjugated anti-IgM (R6-60.2), PE-conjugated anti-IgD (11–26), PE-conjugated anti-Ter119 (Ly-76), PE-conjugated anti-Ly6G and Ly6C (Gr1) (RB6-8C5), Alexa 647 (A647)-conjugated anti-CD8 (53-6.7).

### Mice

We maintained mice on a 12 h light-dark cycle in a temperature-controlled high barrier facility (Monash University ARL) with free access to food and water. *Ptpn2* ^−/−^ mice on a 129sv×BALB/c mixed background have been described previously [25] and were backcrossed onto a BALB/c background for eight generations. *Ptpn2* ^−/−^ (BALB/c) were backcrossed onto a C57BL/6 background for seven generations. *Ptpn2* ^−/−^ (BALB/c) and *Ptpn2* ^−/−^ (C57BL/6) mice were genotyped as described previously [25]. Littermates and aged-matched mice from heterozygous breeding pairs were used in all experiments.

### Generation and genotyping of Ptpn2^ex2−/ex2−^ Mice

*Ptpn2^ex2+/ex2−^* mice were generated by Ozgene (Perth, Australia). The targeting construct was produced using C57BL/6 genomic DNA and the Ozgene PelleNeo vector and incorporated LoxP sites flanking both exon (Ex) 2 and a PGK-Neo-selection cassette flanked by FRT sites inserted downstream of exon 2 (**Fig. 1**). The linearised construct was electroporated into Bruce 4 (C57BL/6J) ES cells and correctly targeted G418 resistant clones identified by Southern blotting and injected into blastocysts for the generation of male chimeras that were mated with C57BL/6J mice to produce *Ptpn2* ***^ex2+^*** ^*/****ex2−***^ offspring. Deletion of exon 2 and removal of the Neo cassette were achieved by crossing with Oz-Cre deleter mice (Ozgene) and the resulting progeny bred with C57BL/6 mice for the elimination of the Cre transgene; deletion of exon 2, removal of Neo and the elimination of Cre were monitored by Southern blotting. *Ptpn2* ***^ex2+^*** ^*/****ex2−***^ mice were genotyped by PCR: forward primer P1 5′TGCAGTTATGGTTTTCCTCAGTCCC3′ and reverse primer P2: 5′ACAGT GCTGGTTGCTGTTTAGCCTC3′.

### Flow cytometry

Freshly isolated thymi, spleens and peripheral lymph nodes were homogenized by gently compressing them between two frosted glass slides or with the sterile end of a plunger and washed with ice-cold PBS supplemented with 2% (v/v) fetal bovine serum (FBS; CSL, Australia); cell suspensions were resuspended further with an 18G needle. Bone marrow was isolated by flushing tibias and femurs with ice cold PBS supplemented with 0.2% BSA with a 26G needle. The resulting marrow was then gently resuspended using a 22G needle. Cell suspensions were recovered by centrifugation (300× g, 5 min at 4°C), red blood cells removed using a red blood cell lysing buffer (Sigma-Aldrich, St Louis, MO) and cell counts (4–15 µm) determined with a Z2 Coulter Counter (Beckman Coulter, Fullerton, CA). For cell surface staining, 3×10^6^ lymphocytes were resuspended in PBS/2% FBS containing the fluorochrome-conjugated antibodies and incubated on ice for 20 min. Cells were washed once in PBS/2% FBS to remove unbound antibodies and analyzed on a LSRII flow cytometer (BD Biosciences, San Jose, CA). For the quantification of red blood cells, mice were bled and a known number of Calibrite™ Beads (BD Biosciences, San Jose, CA) added to a defined volume of diluted blood and analysed by flow cytometry (LSRII). Red blood cells were identified in the forward and side scatter according to their size. Data was analyzed using FACSDiVa (BD Biosciences, San Jose, CA) or FlowJo7 (Tree Star Inc., Ashland, OR) software.

### Histomorphometry

Tibial specimens were fixed in 4% paraformaldehyde/PBS. For histomorphometry, tibiae were embedded in methylmethacrylate [32]. Five µm sagittal sections were stained with toluidine blue, von Kossa stain, and safranin O and histomorphometry was carried out in the secondary spongiosa of the proximal tibia as previously described (OsteoMeasure™, OsteoMetrics, Decatur, GA) [32].

### Body composition

Body composition was measured by DEXA (Lunar PIXImus2; GE Healthcare) and analysed using PIXImus2 software; the head region was excluded from analyses.

### Statistical Analyses

Statistical analyzes were performed using the nonparametric, unpaired Mann-Whitney *U* test and the Long Rank (Mantel-Cox) test using Graphpad Prism (San Diego, CA) software. *P* values of <0.05 were considered significant.

## Supporting Information

Table S1 **Mendelian Analysis of the viability of Ptpn2^ex2−/ex2−^mice.** Progeny from 16 heterozygous breeding pairs were screened by PCR and the Mendelian ratio determined.
(DOCX)

Table S2 **Gross phenotype of Ptpn2^−/−^ (BALB/c), Ptpn2^ex2−/ex2−^ and Ptpn2^−/−^ (C57BL/6) mice.** Runtiness, posture, piloerrection, diarrhoea and eye lid closure were assessed in 18 day-old *Ptpn2^−^* ^/*−*^ (BALB/c), 28 day-old *Ptpn2^ex2−/ex2−^* and 28 day-old *Ptpn2^−^* ^/*−*^ (C57BL/6) mice.
(DOCX)
