# Supplementary material for: Strain-Dependent Differences in Bone Development, Myeloid Hyperplasia, Morbidity and Mortality in Ptpn2-Deficient Mice
Source: PLoS One. 2012 May 8;7(5):e36703. doi: 10.1371/journal.pone.0036703 (PMC3348136; doi:10.1371/journal.pone.0036703)
Supplement: Table S1 — Mendelian Analysis of the viability of Ptpn2ex2−/ex2−mice. Progeny from 16 heterozygous breeding pairs were screened by PCR and the Mendelian ratio determined. (DOCX) [file pone.0036703.s001.docx]

***Table S1. Mendelian Analysis of the viability of Ptpn2^ex2–/ex2–^mice.***

| Parents | *Ptpn2^ex2+/ex–^* x *Ptpn2^ex2+/ex2–^* | | |
| --- | --- | --- | --- |
| Offspring | *Ptpn2^ex2+/ex2+^* | *Ptpn2^ex2+/ex–^* | *Ptpn2^ex2-/ex2–^* |
| Number of animals | 20 | 48 | 20 |
| Mendelian Ratio | 0.23 | 0.54 | 0.23 |

Progeny from 16 heterozygous breeding pairs were screened by PCR and the Mendelian ratio determined.
