# Supplementary material for: Strain-Dependent Differences in Bone Development, Myeloid Hyperplasia, Morbidity and Mortality in Ptpn2-Deficient Mice
Source: PLoS One. 2012 May 8;7(5):e36703. doi: 10.1371/journal.pone.0036703 (PMC3348136; doi:10.1371/journal.pone.0036703)
Supplement: Table S2 — Gross phenotype of Ptpn2−/− (BALB/c), Ptpn2ex2−/ex2− and Ptpn2−/− (C57BL/6) mice. Runtiness, posture, piloerrection, diarrhoea and eye lid closure were assessed in 18 day-old Ptpn2− /− (BALB/c), 28 day-old Ptpn2ex2−/ex2− and 28 day-old Ptpn2− /− (C57BL/6) mice. (DOCX) [file pone.0036703.s002.docx]

***Table S2. Gross phenotype of Ptpn2^–/–^ (BALB/c), Ptpn2^ex2–/ex2–^ and Ptpn2^–/–^ (C57BL/6) mice.***

| **Genotype** | Runtiness | Hunched  posture | Piloerection | Diarrhoea | Eye lid  closure |
| --- | --- | --- | --- | --- | --- |
| *Ptpn2^–^*^/^*^–^* (BALB/c)  n=6 | 6  (100%) | 6  (100%) | 6  (100%) | 6  (100%) | 6  (100%) |
| *Ptpn2^ex2–/ex2–^*  n=8 | 8  (100%) | 0  (0%) | 0  (0%) | 0  (0%) | 0  (0%) |
| *Ptpn2^–^*^/^*^–^* (C57BL/6)  n=8 | 8  (100%) | 0  (0%) | 0  (0%) | 0  (0%) | 0  (0%) |

Runtiness, posture, piloerrection, diarrhoea and eye lid closure were assessed in 18 day-old *Ptpn2^–^*^/^*^–^* (BALB/c), 28 day-old *Ptpn2^ex2–/ex2–^*and 28 day-old *Ptpn2^–^*^/^*^–^* (C57BL/6) mice.
